# Electroconvulsive therapy in bipolar depression – effectiveness and prognostic factors

**DOI:** 10.1111/acps.13075

**Published:** 2019-08-10

**Authors:** K. Popiolek, S. Bejerot, O. Brus, Å. Hammar, M. Landén, J. Lundberg, P. Nordanskog, A. Nordenskjöld

**Affiliations:** ^1^ Faculty of Medicine and Health University Health Care Research Centre Örebro University Örebro Sweden; ^2^ Clinical Epidemiology and Biostatistics Faculty of Medicine and Health Örebro University Örebro Sweden; ^3^ Department of Biological and Medical Psychology University of Bergen Bergen Norway; ^4^ Division of Psychiatry Haukeland University Hospital Bergen Norway; ^5^ Institute of Neuroscience and Physiology The Sahlgrenska Academy at Gothenburg University Gothenburg Sweden; ^6^ Department of Medical Epidemiology and Biostatistics Karolinska Institutet Stockholm Sweden; ^7^ Department of Clinical Neuroscience Center for Psychiatry Research Karolinska Institutet Stockholm Sweden; ^8^ Stockholm County Council Stockholm Sweden; ^9^ Center for Social and Affective Neuroscience Department of Clinical and Experimental Medicine Faculty of Health Sciences Linköping University Linköping Sweden; ^10^ Department of Psychiatry Region Östergötland Linköping Sweden

**Keywords:** bipolar disorders, bipolar depression, electroconvulsive therapy, prognosis

## Abstract

**Objective:**

Electroconvulsive therapy (ECT) is used in patients with severe forms of bipolar depression. ECT is effective but not all patients respond. The aim of this study was to determine prognostic factors for response to ECT in patients hospitalized for bipolar depression.

**Methods:**

Data were obtained from several national Swedish registers. All patients with bipolar depression treated with ECT in any hospital in Sweden between 2011 and 2016 for whom information about ECT response was available were included (*n* = 1251). Response was defined as a score on the Clinical Global Impression – Improvement scale of one or two. Univariate and multivariate logistic regression were conducted to investigate associations between socio‐demographic and clinical factors and response.

**Results:**

Response was achieved in 80.2% patients. Older age was associated with higher response rate to ECT. Patients with comorbid obsessive‐compulsive disorder or personality disorder, and patients previously treated with lamotrigine had lower response rate.

**Conclusion:**

Electroconvulsive therapy for bipolar depression was associated with very high response rates. The strongest prognostic factors were higher age, absence of comorbid obsessive‐compulsive disorder or personality disorder, and less prior pharmacologic treatment.

**Significant outcomes**

- Four of five patients with bipolar depression were responders to electroconvulsive therapy
- The strongest positive prognostic factor for response to ECT was higher age.
- Patients with psychiatric comorbidities, especially personality disorder and OCD had lower chance to respond to ECT.

**Limitations**

- Diagnosis of bipolar depression was most often based on clinical assessment.
- Association between pharmacological treatment and response to ECT might be influenced by indication bias.
- Polypharmacy in majority of patients and dosage of pharmacological agents could have affected associations between pharmacological treatment and outcome.

## Introduction

Electroconvulsive therapy (ECT) is an effective treatment option for patients that suffer from severe depression with response rates of 65.8–80% 1, 2, 3, 4. In clinical practice, ECT is used in both major depressive disorder (MDD) and bipolar depression although the strength of evidence for bipolar depression is lower. Most studies that investigated efficacy of ECT in bipolar depression appear to show no inferiority of ECT in response and remission rates when compared with major depressive disorder 3, 4, 5, 6, 7, but there are reports of bipolarity being a prognostic factor of nonresponse to ECT 1, 8. Clinical guidelines recommend ECT for patients with MDD and bipolar depression who did not respond to pharmacological therapies and in life threatening cases 9, 10, 11, 12. However, not all patients respond to ECT, some others have side effects. Hence, it is important to identify patients who benefit from this treatment. The prognostic factors with most convincing evidence are presence of psychotic features 1, 13, 14 and shorter episode duration 1, 5, 15, yet not all studies confirm these findings 2, 16. Reports concerning other prognostic factors such as age, insufficient response to antidepressants, melancholic features, and symptom severity are more inconsistent. An association between age and response to ECT is relevant as pharmacological treatment in elderly depressed patients are sometimes contraindicated for its side effects. Some studies on patients with unipolar or mixed uni‐ and bipolar samples indicate that older age is a prognostic factor for better ECT efficacy 5, 13, 14, 17. In other studies, however, age had no significant effect on response to ECT 18. Pharmacotherapy resistance constitutes nowadays the main indication for ECT, but the influence of baseline medication on response to ECT is unclear. Studies investigated almost exclusively the role of treatment with antidepressants prior to ECT in patients with MDD. Here, previous studies indicate that resistance to antidepressant medication is predictive for inferior response to ECT 19. Nevertheless, a few newer studies find no relation between resistance to adequate pharmacotherapy and ECT outcome 20, 21, 22. One prospective study investigated the influence of failure of adequate treatment with TCAs and lithium on ECT outcomes and found no such association 22.

Better understanding of factors that predict higher response to ECT including influence of baseline medication on ECT outcome may lead to better assessment of patients with depression. In particular, there is need of more evidence on the effects among patients with bipolar depression.

### Aims of the study

The aim of this study was to examine prognostic factors for response to electroconvulsive therapy in bipolar depression. Also, this study investigated correlations between medication at baseline and response to electroconvulsive therapy. To our knowledge no previous study provides this information.

## Material and methods

### Design

This was a register‐based observational study. For the purposes of the study, data from several national registers were compiled. All patients with bipolar depression treated with ECT and assessed with the Clinical Global Impression – Improvement scale (CGI‐I) 23 were included. A number of clinical variables were selected as possible prognostic factors for ECT response. Univariate and multivariate analyses were conducted to determine associations between variables and response to ECT.

### Participants

All patients in Sweden admitted to a hospital with bipolar depression and treated with ECT between 2011 and 2016 were considered for inclusion in this study (*n* = 1509). CGI‐I score was missing for 258 patients, and these were excluded from the study. In total, 1251 patients entered the study. For patients that were hospitalized more than once during the study period, only the first admission was included in this study.

### Data sources

Data from several national registers were compiled by Statistics Sweden using personal identify number. The Swedish National Patient Register is a mandatory nationwide register of all admissions and outpatient care 24. It provides information on diagnoses and procedures. Medical conditions are coded according to the International Statistical Classification of Diseases and Related Health Problems – Tenth Revision. The Swedish National Quality Register for ECT (Q‐ECT) is a nationwide register that collects detailed data about ECT in Sweden. It is a non‐mandatory register with 89% coverage in 2016 25. The Longitudinal Integration Database for Health Insurance and Labour Market Studies includes all Swedish residents aged 16 or more and provides detailed information about socio‐economic status including family, employment, education, and income 26. The Swedish Prescribed Drug Register provides information about all prescribed medicines that are collected at any pharmacy in Sweden 27.

### Variables

Individuals hospitalized for bipolar depression and receiving ECT were identified from the Swedish National Patient Register (diagnoses F31.3‐5). Information about response to ECT was obtained from the Q‐ECT. Response was quantified using CGI‐I score assessed within 1 week after the completion of ECT. CGI‐I is a seven‐point rating scale that measures the efficacy of treatment. Ratings of one and two indicate “very much improved” and “much improved” respectively. In this study, response was defined as CGI‐I score of one or two. Information about ECT settings and remission was also obtained from the Q‐ECT. Remission was quantified using the Montgomery‐Åsberg Depression Rating Scale Self rated variant (MADRS‐S) 28, 29 score assessed within 1 week after the completion of ECT and was defined as MADRS‐S score <10.

Classification of depressive episode in respect of severity was made according to ICD‐10 diagnosis (F31.3, mild to moderate; F31.4, severe without psychotic symptoms; F31.5, severe with psychotic symptoms). Information about comorbid psychiatric conditions was obtained from the Swedish National Patient Register. Information about level of education and cohabiting was obtained from the Longitudinal Integration Database for Health Insurance and Labour Market Studies. Data describing socio‐economic status the year before admission were used when available. Information on level of education the year before admission was missing for eight patients. For two of these eight patients, information on level of education was available for the year of admission, and this was used instead. The remaining six patients were imputed to the largest category. The term “cohabiting” was defined as living with a partner regardless of marital status and/or living with children.

Information about pharmacological treatment prior to index admission was obtained from the Swedish Prescribed Drug Register. Only medicines that were collected within 100 days before the admission were considered. Psychopharmacological agents were divided into the following groups: lithium, lamotrigine, valproate, quetiapine, antidepressants, antipsychotics (all neuroleptics except quetiapine, alimemazine, levomepromazine), anxiolytics (hydroxyzine, promethazine, alimemazine), benzodiazepines, and central stimulants.

### ECT settings

Electroconvulsive therapy was usually administered three times a week using the bidirectional constant‐current brief‐pulse Mecta (Mecta Corp, Lake Oswego, OR, USA) or Thymatron (Somatics Inc., Lake Bluff, IL, USA) devices. The mean number of ECT treatments was 7.0 (standard deviation [SD], 3.57; range, 1–38). The electrode application during the first ECT treatment was unilateral for 1141 patients (91.2%), bitemporal for 77 patients (6.2%), bifrontal for 27 patients (2.2%), and not known (data missing) for the remaining six patients. The mean pulse width in the first ECT treatment was 0.5 ms (SD, 0.19 ms), the mean frequency was 70 Hz (SD, 21 Hz), the mean duration was 7.3 s (SD, 1.3 s), the mean current was 838.8 mA (SD, 56.4 mA), and the mean charge was 357 mC (SD, 155 mC).

### Statistics

Logistic regression was used to analyze the association between variables and response to ECT. Age was categorized to identify any non‐linear association with response and was analyzed as continuous variable as well. Analyses were conducted using both univariate and multivariate models. Variables included in multivariate models were as follows: gender, age, level of education, cohabiting, severity of depression, anxiety disorder, substance abuse disorder, personality disorder, obsessive‐compulsive disorder (OCD), attention deficit hyperactivity disorder, autism, history of mania, and psychopharmacological treatment prior to admission. Statistical analyses were performed using SAS 9.4 (SAS Institute, Cary, NC, USA) and SPSS 22 (IBM Corp, Armonk, NY, USA).

### Ethics statement

The study was approved by the regional ethical review board in Uppsala 2014/174. Because this was a register‐based study where patients were not identifiable at any time, patients were not informed of the study and were not asked to provide consent.

## Results

### Participants

Socio‐demographic and clinical characteristics of the study cohort are presented in Table 1. Of the 1251 patients included in the study, 830 (66.3%) were women. The mean age of the cohort was 52.5 years, median was 53 years (minimum 16 years, maximum 95 years). Most patients (*n* = 593; 47.4%) had high school education, 246 (19.7%) had less than high school education, and 236 (18.9%) had more than 3 years of college education. The majority of patients (*n* = 639; 51.1%) were living alone. Psychiatric comorbidities were common: 319 patients (25.5%) were diagnosed with substance use disorder, 313 (25.0%) with anxiety disorder, and 187 (14.9%) with personality disorder. Comorbid OCD, attention deficit hyperactivity disorder, and autism were less frequent.

**Table 1:** Socio‐demographic and clinical characteristics of the cohort

	All patients *n* = 1251
	*n* (%)
Gender	
Male	421 (33.7)
Female	830 (66.3)
Age (mean, SD)	52.48 (SD 17.04)
Education	
Less than high school	246 (19.7)
High school	593 (47.4)
College < 3 years	176 (14.1)
College ≥ 3 years	236 (18.9)
Cohabiting	
Yes	612 (48.9)
No	639 (51.1)
Severity of depression	
Mild or moderate	387 (30.9)
Severe without psychotic symptoms	642 (51.3)
Severe with psychotic symptoms	222 (17.7)
Comorbidity	
Anxiety disorder	313 (25.0)
OCD	33 (2.6)
Personality disorder	187 (14.9)
ADHD	68 (5.4)
Autism spectrum disorder	22 (1.8)
Substance abuse disorder	319 (25.5)
Admission due to mania prior to index admission	363 (29.0)
ECT treatment prior to index ECT	
Yes	537 (42.9)
No	329 (26.3)
Missing	385 (30.8)
Pharmacotherapy before admission	
Antidepressants	836 (66.8)
Lithium	473 (37.8)
Benzodiazepines	604 (48.3)
Antipsychotics	541 (43.2)
Anxiolytics	406 (32.5)
Lamotrigine	348 (27.8)
Quetiapine	326 (26.1)
Valproate	170 (13.6)
Central stimulants	43 (3.4)

ADHD, attention deficit hyperactivity disorder; ECT, electroconvulsive therapy; OCD, obsessive‐compulsive disorder; SD, standard deviation.

### Psychopharmacological treatments within 100 days prior to ECT

Antidepressants were used in the 100 days period prior to index admission by 66.8% of patients. Lithium was used by 37.8%, lamotrigine by 27.8%, and valproate by 13.6%. Quetiapine was used by 26.1% of patients, and other neuroleptics were used by 43.2%. Benzodiazepines were used by 48.3% of patients, and anxiolytics by 32.5%. Central stimulants were used by 3.4% of patients.

### Response rate and remission

Of 1251 patients in the study cohort, 1003 (80.2%; 95% confidence interval [CI], 77.8–82.2%) were responders to ECT (CGI‐I score of one or two). Patients assessed as CGI‐I 1 (very much improved) and CGI‐I 2 (much improved) constituted 24.4% and 55.8% of total study population respectively. Symptom worsening, quantified as a CGI‐I score of five, six, or seven (minimally, much, or very much worse) within 1 week after ECT, occurred in eight patients (0.6%). MADRS‐S score within 1 week after ECT was available in 606 patients. Of these, 223 (36.8%; 95% CI, 33.2%‐40.8%) achieved remission (MADRS‐S score: 0–9) and 301 (49.7%) had MADRS‐S score 0–12.

### Prognostic factors

**Figure 1:**
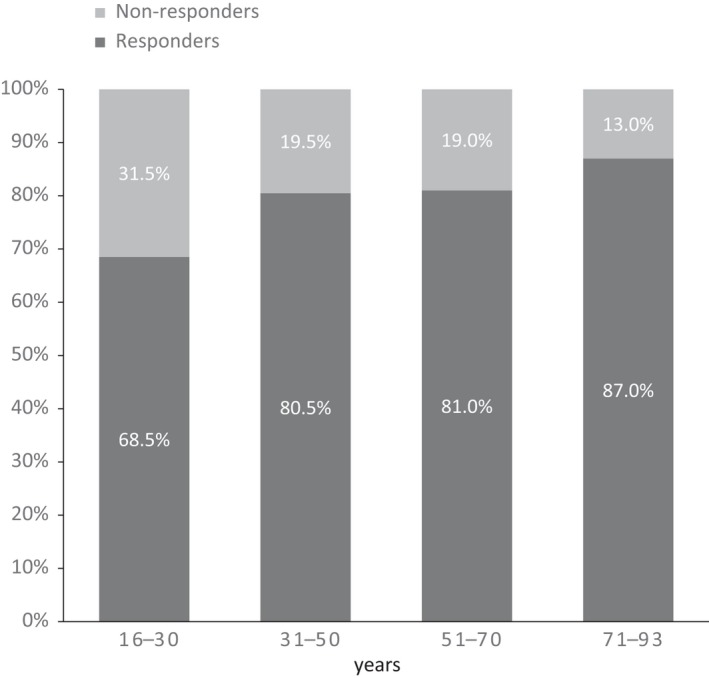
Response to ECT in different age categories expressed as a proportion of patients in each age category.

Associations between socio‐demographic and clinical characteristics and response to ECT are summarized in Table 2. In both univariate and multivariate analysis, patients aged 31–40 years, 61–70 years, or 71‐80 years had higher response rates than patients aged 16–30 years (OR, 2.30; 95% CI, 1.37–3.86; OR, 2.36; 95% CI, 1.47–3.78; and OR, 2.19; 95% CI, 1.15‐4.17 respectively). In univariate analysis, patients in all age categories had significantly higher response rates than patients aged 16–30 years (Fig. 1). Association between age as a continuous variable and outcome was analyzed, as well. This analysis showed a significant relationship between age and response to ECT in both univariate (OR, 1.02 95% CI, 1.01–1.03; *P* = 0.000) and multivariate models (OR, 1.011; 95% CI, 1.001–1.021; *P* = 0.034). Patients with college education longer than 3 years had a significantly higher response rate to ECT in univariate (OR, 1.75; 95% CI, 1.08–2.83; *P* = 0.022) but not multivariate analysis (OR, 1.54; 95% CI, 0.90–2.61; *P* = 0.113). Patients with severe depression with psychotic symptoms had a higher response rate to ECT (OR, 1.73; 95% CI, 1.11–2.72; *P* = 0.016) than patients with mild or moderate depression; however, this association was not significant in the multivariate analysis. Most investigated psychiatric comorbidities were negative prognostic factors. Comorbid personality disorder or OCD remained a significant negative prognostic factor in the multivariate analysis. Among patients with comorbid personality disorder and comorbid OCD 66.3% respective 54.5% were responders to ECT. Patients treated with lamotrigine before index admission had lower response rates to ECT than patients not treated with lamotrigine. This association remained significant in the multivariate model. Patients treated with antidepressants, antipsychotics, benzodiazepines, or anxiolytics had significantly lower response rates than patients not treated with these medications. However, these associations were no longer significant in the multivariate model. There were no significant associations between response and prior treatment with lithium, valproate, quetiapine, or central stimulants. There was no significant association between response and gender, cohabitation, history of mania, or history of treatment with ECT.

**Table 2:** Results for the logistic regression analysis of predictors of good or very good response to ECT for bipolar depression

	*n* (%)	Univariate		Multivariate	
		OR (95% CI)	*P*‐value	OR (95% CI)	*P*‐value
Gender					
Male	421 (33.7)	Reference			
Female	830 (66.3)	1.06 (0.79–1.42)	0.703	1.22 (0.89–1.67)	0.221
Age, years					
16–30	165 (13.2)	Reference			
31–40	174 (13.9)	2.30 (1.37–3.86)	**0.002**	2.06 (1.18–3.60)	**0.011**
41–50	227 (18.1)	1.67 (1.06–2.64)	**0.027**	1.55 (0.95–2.55)	0.082
51–60	240 (19.2)	1.66 (1.06–2.61)	**0.026**	1.48 (0.90–2.42)	0.125
61–70	245 (19.6)	2.36 (1.47–3.78)	**0.000**	1.96 (1.15–3.35)	**0.014**
71–80	150 (12.0)	3.17 (1.77–5.68)	**0.000**	2.19 (1.15–4.17)	**0.018**
81–95	50 (4.0)	2.83 (1.19–6.71)	**0.018**	2.33 (0.91–5.94)	0.077
Education					
Less than high school	246 (19.7)	Reference			
High school	593 (47.4)	1.07 (0.75–1.54)	0.711	1.16 (0.78–1.73)	0.452
Some college < 3 years	176 (14.1)	0.88 (0.55–1.39)	0.574	0.94 (0.57–1.55)	0.815
College>=3 yrs	236 (18.9)	1.75 (1.08–2.83)	**0.022**	1.70 (1.02–2.84)	0.042
Cohabiting					
No	639 (51.1)	Reference			
Yes	612 (48.9)	1.28 (0.97–1.70)	0.081	0.80 (0.59–1.09)	0.153
Severity of depression†					
Mild or moderate	387 (30.9)	Reference			
Severe without psychotic symptoms	642 (51.3)	1.09 (0.80–1.48)	0.593	1.07 (0.77–1.47)	0.700
Severe with psychotic symptoms	222 (17.7)	1.73 (1.11–2.72)	**0.016**	1.43 (0.89–2.29)	0.137
Substance abuse disorder					
No	932 (74.5)	Reference			
Yes	319 (25.5)	0.69 (0.51–0.94)	**0.017**	0.95 (0.67–1.35)	0.781
Anxiety disorder					
No	938 (75.0)	Reference			
Yes	313 (25.0)	0.56 (0.418–0.762)	**0.000**	0.82 (0.58–1.16)	0.259
Personality disorder					
No	1064 (85.1)	Reference			
Yes	187 (14.9)	0.41 (0.29–0.58)	**0.000**	0.59 (0.40–0.87)	**0.008**
OCD					
No	1218 (97.4)	Reference			
Yes	33 (2.6)	0.28 (0.14–0.57)	**0.000**	0.35 (0.16–0.76)	**0.008**
ADHD					
No	1183 (94.6)	Reference			
Yes	68 (5.4)	0.57 (0.33–0.98)	**0.044**	0.84 (0.41–1.72)	0.631
Autism spectrum disorder					
No	1229 (98.2)	Reference			
Yes	22 (1.8)	0.43 (0.18–1.02)	0.056	0.93 (0.34–2.51)	0.882
Admission for mania prior to index ECT‡					
No	888 (71.0)	Reference			
Yes	363 (29.0)	1.00 (0.74–1.36)	0.995	0.99 (0.70–1.40)	0.937
ECT treatment prior to index ECT					
Yes	537 (42.9)	Reference	0.942	1.20 (0.77–1.64)	0.561
No	329 (26.3)	0.99 (0.70–1.39)			
Missing	385 (30.8)				
Psychopharmacotherapy prior to index admission					
Lithium					
No	778 (62.2)	Reference			
Yes	473 (37.8)	1.084 (0.813–1.447)	0.582	1.20 (0.88–1.63)	0.264
Lamotrigine					
No	903 (72.2)	Reference			
Yes	348 (27.8)	0.636 (0.473–0.855)	**0.003**	0.70 (0.51–0.96)	**0.027**
Antipsychotics					
No	710 (56.8)	Reference			
Yes	541 (43.2)	0.697 (0.528–0.922)	**0.011**	0.75 (0.56–1.02)	0.069
Valproate					
No	1081 (86.4)	Reference			
Yes	170 (13.6)	0.838 (0.566–1.238)	0.374	0.91 (0.59–1.38)	0.640
Benzodiazepines					
No	647 (51.7)	Reference			
Yes	604 (48.3)	0.692 (0.523–0.915)	**0.010**	0.80 (0.59–1.09)	0.158
Antidepressants					
No	415 (33.2)	Reference			
Yes	836 (66.8)	0.646 (0.473–0.883)	**0.006**	0.72 (0.51–1.01)	0.059
Anxiolytics					
No	845 (67.5)	Reference			
Yes	406 (32.5)	0.724 (0.542–0.966)	**0.028**	0.95 (0.69–1.30)	0.742
Quetiapine					
No	925 (73.9)	Reference			
Yes	326 (26.1)	0.770 (0.567–1.046)	0.094	0.87 (0.62–1.20)	0.400
Central stimulants					
No	1208 (96.6)	Reference			
Yes	43 (3.4)	0.557 (0.286–1.085)	0.085	1.12 (0.47–2.65)	0.796

Bold values represent *P*‐values <0.05.

ADHD, attention deficit hyperactivity disorder; CI, confidence interval; ECT, electroconvulsive therapy; OCD, obsessive‐compulsive disorder; OR, odds ratio.

^†^According to International Statistical Classification of Diseases and Related Health Problems – Tenth Revision (ICD‐10) diagnoses F31.3–F31.5.

^‡^Diagnoses F30.1, F30.2, F30.9, F31.1, F31.2 according to ICD‐10.

## Discussion

In this study, four of five patients are responders to ECT, a result that supports ECT as an effective treatment method for bipolar depression. Furthermore, only eight patients (0.6%) deteriorated in their psychiatric symptoms after ECT (defined as CGI‐I score 5–7). The latest meta‐analysis on this topic 4 reported a pooled response rate to ECT in patients with bipolar depression 77.1% which is consistent with our study.

In this study, several variables were analyzed as the potential prognostic factors for response to ECT. Both univariate and multivariate models are presented (Table 2). The purpose of that is to present how the model was constructed and to give descriptions of the relationships between variables and response to ECT in univariate models. With the intention of minimizing the effect of confounders we focus on variables that showed significant association with response to ECT in both uni‐ and multivariate models as the most convincing prognostic factors.

The strongest positive prognostic factor for favorable response to ECT was higher age. Earlier studies are inconsistent. van Diermen et al. 14 showed that ECT is more effective in patients at higher age. Haq et al. reported an association between higher age and better response to ECT in their meta‐analysis, but dispute the clinical relevance of this observation. Birkenhäger et al. reported no association between response to ECT and age as a continuous variable, but the youngest age group (18–45 years) had the lowest rate of response. The size of study cohorts and differences in statistical analysis may contribute to the inconsistencies. Comparisons with other studies are difficult as other study cohorts included both unipolar and bipolar depression.

In the current study, comorbid psychiatric disorders were associated with reduced likelihood of responding to ECT. Patients with personality disorder or OCD were significantly less likely to respond to ECT in both univariate and multivariate models. Few studies have investigated the role of comorbidities on ECT outcome. Some indicate that comorbid substance use disorders or personality disorders may negatively affect the outcome of ECT 30, 31. Medda et al. 1 reported poorer outcomes for patients with comorbid panic disorder – agoraphobia but not comorbid social phobia, OCD, or alcohol or drug abuse, but included few patients with comorbidities and the study therefore had limited power to detect differences in outcomes. Our observation that patients with comorbidities had a lower response rate to ECT is in line with studies showing poorer outcomes of medication treatment in patients with psychiatric comorbidities 32, 33.

We investigated the impact of medication prior to ECT and observed that patients treated with lamotrigine had significantly lower response rate than those without prior treatment with lamotrigine. One possible explanation is that pharmacological treatment prior to ECT reflects physicians’ efforts to alleviate depressive symptoms. Presumably, pharmacological treatment is an indicator for therapy refractory depression in this study population. These results suggest that resistance to pharmacological treatment might be a risk factor for lower response rate to ECT. There is also a possibility that continued use of lamotrigine during ECT might decrease the quality of the seizure, which might also lower the response rate. The impact of medication prior to ECT on response needs further investigations.

Psychotic symptoms were not significantly associated with response to ECT after adjustment to other variables in this study. Previous studies are equivocal as to whether or not presence of psychotic symptoms is an independent predictor of response to ECT. The results may have been confounded by the duration of depressive episode. In the current study, the duration of the depressive episode was not investigated. More studies are needed to evaluate the role of psychosis as a prognostic factor for the outcome of ECT.

In this study 37% of patients achieved remission. Remission rates reported by other investigators vary between 43 and 87% 4, 34. Discrepancies between studies can be because of differences in study populations and design. Another possible explanation might be ECT settings. In this study, 91% of patients were treated with right unilateral electrode placement. Superiority of bilateral electrode placement has been suggested by some studies 35. Yet, a recent meta‐analysis failed to demonstrate significant difference in antidepressant efficacy between high‐dose unilateral and bilateral ECT 36. Another issue worth consideration is number of treatment sessions. The mean number of sessions in this study was seven. Husain et al. 37 investigated speed of response and showed that 34% of patients achieved remission after six treatments and 65% achieved remission after 10 treatments. This point to longer treatment series could be more beneficial for selected patients. Also, brief pulse width has been suggested as more effective than ultrabrief 38. In this study the mean pulse width was 0.5 ms. It is possible that more optimal use of ECT, than current Swedish clinical practice, could result in higher remission rates.

In this study, 66.3% of patients treated with ECT were women. This is consistent with other studies. Although there is no difference in the prevalence of bipolar disorder between men and women, women are over‐represented in studies of ECT, probably because of the burden of depressive symptoms. Women with bipolar disorder have more depressive episodes, usually with longer duration 39.

Among patients considered as participants in this study, 258 were excluded because of missing CGI‐I value. That represent patients that were not included in the Q‐ECT. It is possible that these patients had lower response rate than study population, because centers that include a higher proportion of patients in the Q‐ECT may also provide higher quality of service. It could result in overestimating the effectiveness of ECT.

The present study has several limitations. In most cases, the diagnosis was based on clinical assessment, and it is not known whether or not structured diagnostic methods were used. The information about dosages of pharmacological agents is lacking. The associations between pharmacological treatment and response to ECT may be influenced by indication bias. Multiple adjustments for other variables such as severity of symptoms and presence of psychotic symptoms was done to limit this indication bias. Many patients had been treated with several medicines, and possible interactions between them were not taken into consideration. On the other hand, our findings are strengthened by the large group of patients in the relatively homogenous clinical settings all through Sweden and thus provide a guidance to the clinician's decision making regarding ECT.

In conclusion, the present study shows that ECT for bipolar depression was associated with very high response rates. The strongest prognostic factors for better outcome were higher age and the absence of psychiatric comorbidities. This study suggests that resistance to lamotrigine but not to other pharmacological agents might predict lower response rate. Associations between pharmacotherapy prior to ECT and response to ECT need further investigation.

## Conflicts of interest

None.

## Data Availability

There are no linked research data sets for this submission. The following reason is given: The data that has been used is confidential.
